# Strong expression of polypeptide *N*-acetylgalactosaminyltransferase 3 independently predicts shortened disease-free survival in patients with early stage oral squamous cell carcinoma

**DOI:** 10.1007/s13277-015-3928-7

**Published:** 2015-08-22

**Authors:** Yoshikazu Harada, Hiroto Izumi, Hirotsugu Noguchi, Akihiro Kuma, Yuichiro Kawatsu, Tomoko Kimura, Shohei Kitada, Hidetaka Uramoto, Ke-Yong Wang, Yasuyuki Sasaguri, Hiroshi Hijioka, Akihiko Miyawaki, Ryoichi Oya, Toshiyuki Nakayama, Kimitoshi Kohno, Sohsuke Yamada

**Affiliations:** 1 Department of Pathology, School of Medicine, University of Occupational and Environmental Health, 1-1 Iseigaoka, Yahatanishi-ku, Kitakyushu, 807-8555 Japan; 2 Department of Dentistry and Oral Surgery, University Hospital of Occupational and Environmental Health, Kitakyushu, 807-8555 Japan; 3 Department of Occupational Pneumology, University of Occupational and Environmental Health, Kitakyushu, 807-8555 Japan; 4 Department of Second Internal Medicine, University of Occupational and Environmental Health, Kitakyushu, 807-8555 Japan; 5 Department of Health Policy and Management, Institute of Industrial Ecological Sciences, University of Occupational and Environmental Health, Kitakyushu, 807-8555 Japan; 6 Department of Urology, University of Occupational and Environmental Health, Kitakyushu, 807-8555 Japan; 7 Second Department of Surgery, University of Occupational and Environmental Health, Kitakyushu, 807-8555 Japan; 8 Shared-Use Research Center, School of Medicine, University of Occupational and Environmental Health, Kitakyushu, 807-8555 Japan; 9 Laboratory of Pathology, Fukuoka Wajiro Hospital, Fukuoka, 811-0213 Japan; 10 Department of Oral and Maxillofacial Surgery, Field of Oral and Maxillofacial Rehabilitation, Advanced Therapeutics Course, Graduate School of Medical and Dental Sciences, Kagoshima University, Kagoshima, Kagoshima 890-8520 Japan; 11 Asahi-Matsumoto Hospital, Kitakyushu, 800-0242 Japan; 12 Institute of Pathology, Medical University of Graz, Graz, 8010 Austria; 13 Institute of Molecular Biosciences, University of Graz, Graz, 8010 Austria

**Keywords:** Oral squamous cell carcinoma (OSCC), Polypeptide *N*-acetylgalactosaminyltransferase 3 (GalNAc-T3), Early stage OSCC (ESOSCC), Vessel invasion, Disease-free survival (DFS)

## Abstract

The polypeptide *N*-acetylgalactosaminyltransferase (GalNAc-Ts) family of enzymes regulates the critical initial steps of mucin-type *O*-glycosylation. Among GalNAc-Ts that may significantly influence cancer biology, thus affecting cell differentiation, adhesion, invasion, and/or metastasis, GalNAc-T3 exhibits a high expression in several human cancers, closely associated with tumor progression and a poor prognosis. However, the expression pattern of GalNAc-T3 in oral squamous cell carcinoma (OSCC) remains obscure. Since postoperative recurrence of even early stage OSCC (ESOSCC) occurs at an early phase, significantly affecting their clinical course and worse outcome, the identification of clinically significant accurate biomarkers is needed. Therefore, we investigated the correlation between the immunohistochemical GalNAc-T3 expression and various clinicopathological characteristics and recurrence using 110 paraffin-embedded tumor samples obtained from patients with surgically resected ESOSCC (T1–2N0). Recurrence was recognized in 37 of 110 (33.6 %) patients. The GalNAc-T3 expression was considered to be strongly positive when 20 % or more of the cancer cells showed positive cytoplasmic staining. Consequently, a strong expression of GalNAc-T3 was observed in 40 patients (36.4 %), showing a close relationship to poor differentiation, the presence of lymphatic and vascular invasion, and recurrence. Univariate and multivariate analyses further demonstrated that the patients with a strong GalNAc-T3+ status had markedly lower disease-free survival (DFS) rates, especially within the first 2 years postoperatively. Therefore, GalNAc-T3 might play a role in the pathogenesis of ESOSCC recurrence, and its immunohistochemical detection potentially predicts a shorter DFS and may be a useful parameter for providing clinical management against ESOSCC in the early postoperative phase.

**Electronic supplementary material:**

The online version of this article (doi:10.1007/s13277-015-3928-7) contains supplementary material, which is available to authorized users.

## Introduction

Oral cancer is the most common head and neck cancer, which is the sixth most common malignancy worldwide, including in Japan. Currently, approximately 264,000 new cases are diagnosed each year, with oral squamous cell carcinoma (OSCC) comprising more than 90 % histological types of these cases. Of these cases, more than 128,000 patients die of the disease annually, and the mortality rate of OSCC is 3.7 per 100,000 in Japan alone [1–3]. Among various clinicopathological characteristics, local recurrence and/or regional lymph node metastasis in cases of postoperative OSCC in particular have been proposed to be prognostic indicators [4–6]. Even early stage OSCC (ESOSCC) lesions treated with surgery alone, which more than 80 % can be cured by therapy, may exhibit postoperative relapse within the first 2 to 3 years [6–8]. Therefore, it is critical to predict which ESOSCC patients (T1–2N0) are prone to recurrence/metastasis and mortality after surgery using practically accurate biomarkers. The clinical picture of OSCC is also significantly determined by the complex interplay among additional cellular alterations, e.g., epigenetic modulation of the gene expression, at least in part [1, 4].

It is well known that the malignant transformation and cancer progression have a close relationship with alterations in cell surface carbohydrate antigens (CAs), as well as frequent aberrant glycosylation [9]. Glycosylation, a major type of post-translational modification of most secretory and cellular proteins, is capable of altering their physicochemical properties and biological activities, leading to change the functions of glycoproteins and transform cellular phenotypes, especially in cancer.

*O*-glycosylation reportedly encompasses diverse classes of glycoproteins and, particularly, mucin-type *O*-linked glycans constitute up to 80 % of the total amount of CAs in mammals [9–11]. Mucin-type *O*-glycosylation is initiated by uridine diphosphate (UDP) *N*-acetyl-α-d-galactosamine polypeptide (GalNAc) *N*-acetylgalactosaminyltransferases (GalNAc-Ts). GalNAc-Ts can catalyze the transfer of GalNAc from the sugar donor UDP-GalNAc to serine and threonine residues on protein synthesizing CAs in the Golgi apparatus and are the largest glycosyltransferase enzyme families covering a single known glycosidic linkage [12–14]. Abnormal *O*-linked glycans expressed in many epithelial cancers are markedly associated with the presentation of incompletely elongated and shortened glycan structures, which may affect cell differentiation, adhesion, invasion, and/or metastasis [10, 11]. The GalNAc-Ts, classified as 27 family members and, to date, consisting 24 members in human, show a tissue-specific expression and have different kinetic properties and acceptor substrate specificities [14]. Many studies, including ours, have indicated that GalNAc-T3 should be significantly useful for evaluating various types of carcinoma, including digestive tract adenocarcinoma, lung adenocarcinoma, renal cell carcinoma, prostatic adenocarcinoma, and ovarian adenocarcinoma [11, 15–24]. In contrast, there are no studies of possible associations between the GalNAc-T3 expression in OSCC, especially ESOSCC, and associated clinicopathological features, such as patterns of tumor invasiveness, vessel permeation, or the patient’s prognosis.

In the present study, we show for the first time that, in patients with postoperative ESOSCC, a cytoplasmic strong GalNAc-T3 expression is significantly correlated with a shortened disease-free survival (DFS) and that this molecule might be a promising biomarker for the clinical management of ESOSCC.

## Materials and methods

### Patients

All intended procedures in the present study, including the use of specimens obtained from human subjects, were approved by the Ethics Committee of the University of Occupational and Environmental Health (UOEH) in Kitakyushu, Japan (permission number H27-004), as well as written consent of the patient’s next of kin according to the guidelines of the Japanese Society of Pathology. Pathological reports were reviewed to identify patients who had undergone surgical resection without neck lymph node dissection for ESOSCC between 1994 and 2015 at the hospital of UOEH.

There were no patients with perioperative death, defined as death during the initial hospitalization or within 30 days of surgery. A total of 110 surgically resected ESOSCC (T1–2N0) patients with available follow-up data comprised the cohort of this retrospective study, as shown in Table 1, after further excluding those with the following characteristics: (a) other prior or concomitant malignant tumors; (b) coexisting medical problems of sufficient disease severity to shorten life expectancy; (c) involvement of the resection margin; and (d) the use of neoadjuvant chemotherapy or radiotherapy prior to surgery. The surgical margins were considered to be involved when the presence of invasive carcinoma and/or carcinoma in situ at the margin of the mucosa was identified, or the distance to the non-carcinomatous mucosa margin was less than 5 mm.

**Table 1:** Clinicopathological characteristics of the patients

Characteristic	Patients (*n* = 110)
Age (years)	
Average	64.3
Median	67
Range	21–92
>60	78
≦60	32
Sex	
Male	61
Female	49
ECOG PS	
≦1	100
≧2	10
Follow-up months	
Average	67.2
Median	56.6
Range	1–236
Tumor location	
Tongue	65
Maxilla	7
Mandible	9
Buccal	10
Floor of mouth	18
Hard palate	1
Tumor size (cm)	
Average	2
Median	2
Range	0.2–3.8
T stage	
T1	59
T2	51
Tumor differentiation	
Well	68
Moderately	30
Poorly	12
Mode of invasion	
1	16
2	28
3	54
4C	9
4D	3
Vascular invasion	
v(−)	101
v(+)	9
Lymphatic vessel invasion	
ly(−)	92
ly(+)	18
Perineural involvement	
ne(−)	107
ne(+)	3

*ECOG PS* Eastern Cooperative Oncology Group performance status, *v* vascular invasion, *ly* lymphatic vessel invasion, *ne* perineural involvement

Clinical information was thoroughly gathered from the patients’ records. The duration of survival (DFS and OS) was defined as the interval from the date of surgery to recurrence and death or the most recent clinic visit, respectively. In this study, “recurrence” (37 cases; 33.6 %) included “local recurrence,” “subsequent regional lymph node metastasis,” and “locoregional recurrence.” “Local recurrence” (13 cases; 11.8 %) was defined as lesions arising in the oral cavity relative to the primary tumor beyond 6 weeks within the first 5 years after the first definitive treatment. Meanwhile, recurrence arising in the neck was defined as “subsequent regional lymph node metastasis” (29 cases; 26.4 %), and recurrence arising at both the primary site and in the neck was defined as “locoregional recurrence” (5 cases; 4.5 %), as shown in Table 2. Patients with recurrence considered to be potentially curable and operable (35 cases; 31.8 %) underwent salvage surgery with radiotherapy (eight cases) or radiotherapy and adjuvant chemotherapy (six cases), as follows: carboplatin (two cases), fluorouracil plus carboplatin (two cases), cetuximab (one case), and docetaxel (one case), respectively. Another two inoperable (1.8 %) patients received radiotherapy and chemotherapy, as follows: fluorouracil plus carboplatin (one case) and cetuximab (one case). All patients were followed up every 2 weeks and 1 month within the first and second postoperative year, respectively, and at approximately 2- to 6-month intervals thereafter using visual inspection of the oral cavity and/or neck CT scans. Chest X-ray was performed every 6 months for 5 years after surgery. Additional examinations, including brain, chest and abdominal CT, MRI, and bone scintigraphy, were performed rarely in cases involving signs or symptoms of recurrence.

**Table 2:** Outcome of the recurrence status in the patients on the end of this study

	Total (*n* = 110)	% of all	% of recurrent group
No recurrence	73	66.4	
Recurrence^a^	37	33.6	
Local recurrence	13	11.8	35.1
Subsequent regional lymph node metastasis	29	26.4	78.4

^a^Locoregional recurrence: *n* = 5

### Tissue specimens

Formalin-fixed, paraffin-embedded tissue blocks were provided by our Department of Pathology. Three pathologists examined all resected specimens to confirm the histopathological features. The stage grouping of the tumors was classified according to the TNM classification of malignant tumors, seventh edition [25]. The mode of invasion was examined at the host/tumor interface as previously defined by Yamamoto et al. [26]. Each patient was assigned an Eastern Cooperative Oncology Group performance status (ECOG PS) score at the time of diagnosis [27]. Non-carcinomatous tissue and recurrent OSCC tissue were obtained from non-tumor portions of the surgically resected specimens and metastatic regional lymph nodes or locally recurrent oral mucosa corresponding to each primary site, respectively. These tissues were stained with hematoxylin and eosin (H&E), Elastica van Gieson (EVG), or immunohistochemistry preparations of sequential sections. EVG, immunohistochemical Podoplanin (D2-40; Nichirei Bioscience Co., Tokyo, Japan; diluted 1:1), and S-100 protein (Dako, Glostrup, Denmark; diluted 1:900) staining very clearly revealed vascular invasion (v), lymphatic vessel invasion (ly), and perineural involvement (ne), respectively, as previously described [28].

### Preparation of antibodies against GalNAc-T3 and other GalNAc-Ts

**Fig. 1:**
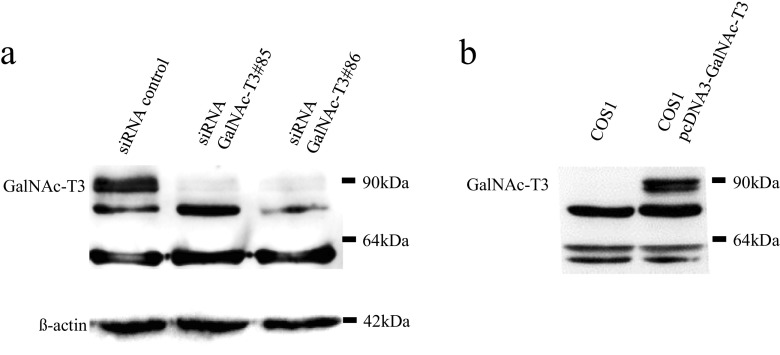
The specificity of our original polyclonal antibody for GalNAc-T3 was confirmed using Western blotting. **a** A Western blotting analysis confirmed the specificity of the GalNAc-T3 antibody. HSC-2 cells were transfected with siRNA control and GalNAc-T3 siRNA knockdown (no. 85 and no. 86), and 30-μg protein from whole cell lysates was applied. β-actin was used as an internal control. **b** COS-1 cells were transfected with GalNAc-T3 expression plasmid, and 30 μg protein from whole cell lysates was applied

A polyclonal antibody was raised against GalNAc-T3 using multiple immunizations of New Zealand white rabbits with a synthetic peptide, based on previously published work (sequence: GYYTAAELKPVLDRPPQDS) [29]. An anti-GalNAc-T6 polyclonal antibody was generated in the same way, as described in a previously published paper (synthetic peptide sequence: GFYTPAELKPFWERPPQDP) [21]. The specificity of our original antibody was confirmed on Western blotting (Fig. 1). Furthermore, we performed immunohistochemistry with peptide competition against GalNAc-T3 [16, 29] and GalNAc-T6 [21], respectively. For immunohistochemistry of GalNAc-T3 (diluted 1:1,000), we used well-differentiated tubular colorectal adenocarcinoma cells as positive controls [11]. To analyze the staining or expression profiles of other GalNAc-Ts, anti-GalNAc-T1, anti-GalNAc-T2, and anti-GalNAc-T4 goat monoclonal antibodies (1:100; Santa Cruz Biotechnology, Santa Cruz, CA, USA) and the above anti-GalNAc-T6 (diluted 1:1,000) were applied, respectively.

### Cell culture

HSC-2 (human OSCC cell line) was described previously [30] and maintained in Eagle’s minimal essential medium containing 10 % fetal bovine serum. COS-1 (monkey kidney cell line) was described previously [31] and maintained in Dulbecco’s modified Eagle’s medium containing 10 % fetal bovine serum. Cell lines were maintained at 37 °C in an atmosphere of 95 % air and 5 % CO_2_.

### GalNAc-T3 siRNA knockdown and overexpression

Twenty-five base-pair double-stranded RNA oligonucleotides were commercially generated (Life Technologies, CA, USA): 5′-UUCUGCUGCUGUAUAAUAUCCUUGC-3′ and 5′-GCAAGGAUAUUAUACAGCAGCAGAA-3′ for GalNAc-T3#85 and 5′-AAACUAUUAUGACACUGGUGGUGGG-3′ and 5′-CCCACCACCAGUGUCAUAAUAGUUU-3′ for GalNAc-T3#86. siRNA transfection was performed as previously described [11]. A total of 5 μL of RNAiMAX (Life technologies) was diluted in 250 μL of Opti-MEM medium (Invitrogen, Carlsbad, CA, USA) and incubated for 5 min at room temperature (RT). Next, 250 pmoL of siRNA for GalNAc-T3 and RNAiMAX as control siRNA diluted in 250 μL of Opti-MEM were gently added and incubated for 20 min at RT. Oligomer-RNAiMAX complexes and aliquots of 2 × 10^5^ HSC-2 in 500 μL of culture medium were combined and incubated. To obtain pcDNA3-GalNAc-T3 expression plasmid, human full length GalNAc-T3 cDNA was amplified with the primer pairs 5′-CCATGGCTCACCTAAAGCGACTAG-3′ and 5′-TTAATCATTTTGGCTAAGTATCC-3′ and ligated in multi-cloning site of pcDNA3 mammalian expression plasmid (Invitrogen). Underlines indicate start or stop codons. Transfection of expression plasmid was described previously [32].

### Western blotting

Transfected cells with siRNAs or expression plasmid were harvested after 48 h, and a Western blotting analysis was performed as previously described [11]. In total, 30 μg of protein from whole cell lysates was separated via sodium dodecyl sulfate-polyacrylamide gel electrophoresis and transferred to polyvinylidene difluoride microporous membranes (Millipore, MA, USA). The blotted membranes were treated with 5 % (*w*/*v*) skim milk and incubated for 1 h at RT with the primary antibody (diluted 1:1,000). Next, the membrane was incubated for 45 min at RT with a peroxidase-conjugated secondary antibody and visualized using an ECL kit (GE Healthcare Bio-Science, Buckinghamshire, UK). Detection was performed with an LAS-4000 mini (Fujifilm, Tokyo, Japan).

### Immunofluorescence of HSC-2

HSC-2 was cultured on coverslips, then incubated with anti-GalNAc-T3 (diluted 1:1,000) and anti-GalNAc-T6 (diluted 1:1,000) rabbit polyclonal antibodies or anti-GalNAc-T1, anti-GalNAc-T2, and anti-GalNAc-T4 goat monoclonal antibodies (diluted 1:100; Santa Cruz Biotechnology) for 1 h at RT, respectively. These cells were then further incubated with Hoechst 33258 (*blue-stained*) (0.5 mg/mL; Dojindo, Kumamoto, Japan) and visualized with goat anti-rabbit or donkey anti-goat IgG antibodies conjugated with Alexa Fluor dyes (*green-stained*), respectively [11]. After being washed with PBS, the specimens were observed under a Nikon ECLIPSE E600 inverted fluorescence microscope (Nikon, Tokyo, Japan).

### Immunohistochemistry of the tissue samples

Immunohistochemical staining was performed according to the antibody-linked dextran polymer method for antibody-bridge labeling, with hematoxylin counterstaining (EnVision; DAKO). Deparaffinized and rehydrated 4-μm sections were incubated in 10 % H_2_O_2_ for 5 min to block the endogenous peroxidase activity. The sections were subsequently rinsed and incubated with rabbit polyclonal anti-GalNAc-T3 (diluted 1:3,000) antibodies for 30 min, respectively. The second antibody-peroxidase-linked polymers were then applied, and the sections were incubated with a solution consisting of 20 mg of 3.3′-diaminobenzidine tetrahydrochloride, 65 mg of sodium azide, and 20 mL of 30 % H_2_O_2_ in 100 mL of Tris–HCl (50 mM, pH 7.6). After counterstaining with Meyer’s hematoxylin, the sections were observed under a light microscope. Each section was first scanned at low power for all fields (original magnification: ×40) using tumor and non-tumor tissues, respectively, in order to determine the heterogeneity of the distribution. The number of positive cells showing cytoplasmic staining was recorded in the site of invasion. Necrotic tissues, stromal cells, and lymphoid cells were not included in the recordings.

The degree of immunoreactivity for GalNAc-T3 was assessed in each case semi-quantitatively by evaluating the proportion of positive cells relative to the total number of ESOSCC cells. Samples with positive areas comprising equal to or more than 20 % of the neoplasms were considered to be strongly positively stained. We selected and validated immunohistochemical cutoff scores for GalNAc-T3 positivity, based on the findings of a receiver operating characteristic (ROC) curve analysis [33]. Finally, all patients were divided into two groups, as follows: those with strongly positive findings, equal to or more than 20 %, and those with weakly positive (including negative) findings, less than 20 %.

All histological and immunohistochemical slides were evaluated by two independent observers using a blind protocol design (the observers were blinded to the clinicopathological data). The degree of agreement between the observers was excellent (an agreement rate of more than 95 %) for all antibodies investigated, as measured according to the interclass correlation coefficient. For the very few (much less than 1 %) instances of disagreement, a consensus score was calculated by a third board-certified pathologist in our department [11, 28, 34, 35].

### Statistical analysis

The significance of correlations was determined based on *χ* ^2^ test or Fisher’s exact test, where appropriate, in order to assess the relationships between the immunohistochemical expression levels and the clinicopathological variables [11, 30]. Survival curves were plotted according to the Kaplan–Meier method and compared using the log-rank test. Hazard ratios and 95 % confidence intervals (95 % CIs) were estimated using univariate or multivariate Cox proportional hazard models. All statistical tests were two-tailed, with a *P* value of <0.05 considered to be statistically significant. All statistical analyses were performed with the EZR software program (Saitama Medical Center, Jichi Medical University, Japan), a graphical user interface for R (The R Foundation for Statistical Computing, version 2.13.0) [35, 36]. More precisely, this program is a modified version of R commander (version 1.6-3) designed to add statistical functions frequently used in biostatistics.

## Results

### Confirmation of the specificity of the GalNAc-T3 antibody

Specificity, a GalNAc-T3 polyclonal antibody, was tested using immunohistochemistry and Western blotting, as previously described [11]. In this study, Western blotting revealed that GalNAc-T3 proteins were expressed in the HSC-2 cells (Fig. 1a), whereas GalNAc-T6 staining was weak (data not shown). We thus performed siRNA transfection of GalNAc-T3 into the wild-type HSC-2 cells, which demonstrated that the expression of about 90 kDa protein was significantly decreased in the HSC-2 cells of GalNAc-T3 siRNA (Fig. 1a). Further, about 90 kDa protein was detected in cells transfected with GalNAc-T3 expression plasmid (Fig. 1b). These Western blotting results indicate that the GalNAc-T3 antibody was specific for its protein. Immunofluorescence staining of the OSCC cell line (HSC-2) showed a specific expression of GalNAc-T3 in a cytoplasmic, perinuclear fashion but not in nuclei (Supplementary Fig. 1). In contrast, other GalNAc-Ts (-T1, -T2, -T4, and -T6) were not or very weakly expressed in the HSC-2 cells (Supplementary Fig. 1).

### Patient characteristics

The cohort included 110 patients (61 males, 49 females) with clinicopathological variables representative of ESOSCC (Table 1). The average age at surgery was 64.3 years. A total of 100 (90.9 %) and 10 (9.1 %) patients had an ECOG status equal to or less than 1 and more than 2, respectively. The median tumor size was 2.0 cm (range = 0.2–3.8 cm). At diagnosis, no patients had either lymph node or distant metastasis. The tumor grade included 68 well-differentiated lesions (61.8 %), 30 moderately differentiated lesions (27.3 %), and 12 poorly differentiated lesions (10.9 %) of squamous cell carcinoma. Based on the (TNM) classification of malignant tumors, seventh edition, the ESOSCC patients had stage I (56/110; 50.9 %) and II (54/110; 49.1 %) disease, respectively. Postoperative follow-up was available for all 110 patients (average = 67.2 months; range = 1–236 months). The median OS was 56.6 months, with 2- and 5-year survival rates of 92.9 and 90.9 %, respectively. Moreover, the median DFS was 26.5 months with 2- and 5-year survival rates of 67.5 and 61.9 %, respectively. Supplementary Table 1 displays each patient’s information in detail.

### GalNAc-T3 expression in the non-carcinomatous oral mucosal tissues, ESOSCC specimens, and recurrent OSCC specimens of lymph nodes or mucosa

**Fig. 2:**
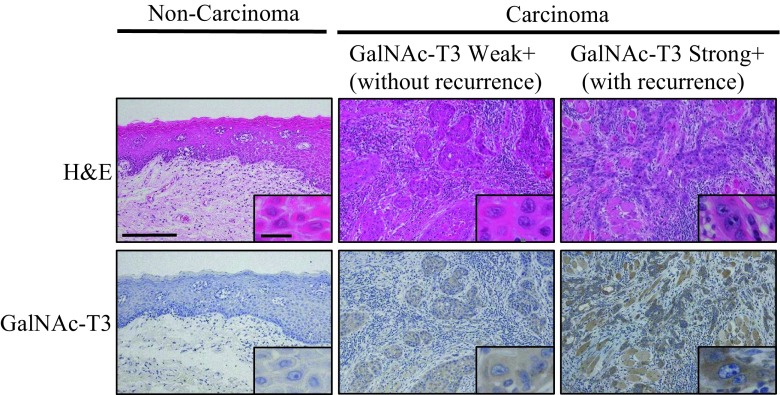
The cytoplasmic GalNAc-T3 expression patterns showed specifically positive staining on immunohistochemistry, potentially associated with early recurrence in the ESOSCC patients. Representative images of immunohistochemically strong/weak positivity for GalNAc-T3 in the human ESOSCC samples are shown, demonstrating a cytoplasmic staining pattern in the strong GalNAc-T3-positive cases with postoperative recurrence (case no. 25), compared to the scant cytoplasmic staining pattern in the weak GalNAc-T3-positive cases without any recurrence (case no. 71) (Original magnification: ×100; *inset*, ×400). In contrast, a cytoplasmic GalNAc-T3 expression was very weakly detectable of adjacent non-carcinomatous squamous epithelium (normal to dysplasia). Each inset provides a representative image of ESOSCC carcinomatous and non-carcinomatous nuclei and cytoplasm on high-power view. *H&E* hematoxylin and eosin. *Bars* = 200 μm (×100) and 20 μm (×400)

**Fig. 3:**
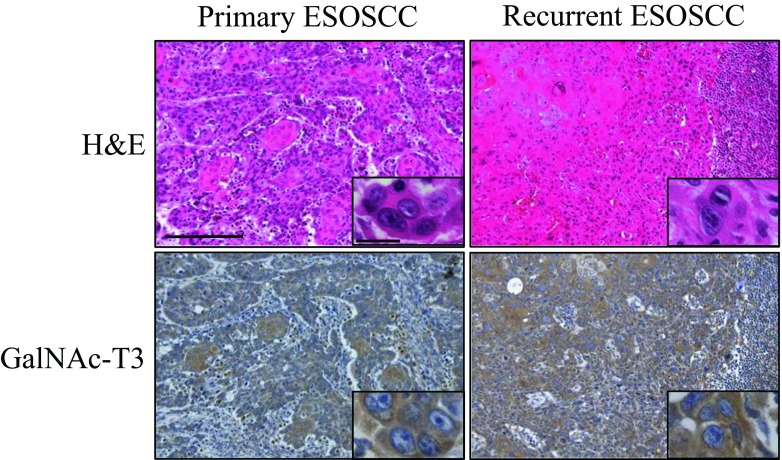
GalNAc-T3 staining patterns significantly corresponding to the primary ESOSCC specimens. Representative immunohistochemical GalNAc-T3 sections are presented with a strong expression of early recurrent ESOSCC in both the primary oral mucosa and metastatic regional lymph node (case no. 21). *H&E* hematoxylin and eosin. *Bars* = 200 μm (×100) and 20 μm (×400)

On immunohistochemistry, GalNAc-T3 showed cytoplasmic expression patterns in the typically strongly positive cancer cases (Fig. 2), especially at the invasive front. Representative images of the immunohistochemical findings of the human ESOSCC samples associated with a shortened DFS (i.e., early recurrence) or no recurrence are shown in Supplementary Table 1 (case nos. 25 or 71), respectively (Fig. 2). In contrast, a cytoplasmic GalNAc-T3 expression was very weakly, but on occasion, diversely, detectable of adjacent non-carcinomatous squamous epithelium (normal to dysplasia) in the paraffin-embedded tissues (Fig. 2). GalNAc-T3 was weakly and strongly positively expressed in 70 (63.6 %) and 40 (36.4 %) of the 110 ESOSCC specimens, respectively (Table 3 and Fig. 2). In particular, GalNAc-T3 was strongly or weakly expressed in 19 (54.3 %) and 16 (45.7 %) of 35 recurrent ESOSCC specimens of lymph nodes or corresponding oral mucosa, respectively. Moreover, the GalNAc-T3 staining patterns were borderline significantly consistent with those of the corresponding primary OSCC specimens (*P* = 0.05), especially the recurrent pattern of subsequent regional lymph node metastasis (Table 2 and Supplementary Table 2), as described later in detail. Representative H&E and immunohistochemical GalNAc-T3 sections are shown with a strong expression of OSCC in both the primary oral mucosa and metastatic regional lymph nodes (case no. 21 in Supplementary Table 1) (Fig. 3).

**Table 3:** Detailed correlations between the GalNAc-T3 expression and the clinicopathological variables

Variables	Total	GaINAc T3 expression			
		(%)	Weak+ (*n* = 70)	Strong+ (*n* = 40)	*P* value
Age					0.05
>60	32	29.1	25 (78.1)	7 (219)	
≦60	78	70.9	45 (57.7)	33 (42.3)	
Sex					0.24
Male	61	55.5	42 (68.9)	19 (31.1)	
Female	49	44.5	28 (57.1)	21 (42.9)	
ECOG PS					0.74
0–1	100	90.9	63 (63.0)	37 (37.0)	
2–4	10	9.1	7 (70.0)	3 (30.0)	
Smoking					0.17
(−)	51	46.4	36 (70.6)	15 (29.4)	
(+)	59	53.6	34 (57.6)	25 (42.4)	
Alcohol					0.43
(−)	49	44.5	29 (59.2)	20 (40.8)	
(+)	61	55.5	41 (67.2)	20 (32.8)	
T stage					0.11
T1	59	53.6	42 (71.2)	17 (28.8)	
T2	51	46.4	28 (54.9)	23 (45.1)	
Tumor location					0.32
Tongue	65	59.1	39 (60.0)	26 (40.0)	
Maxilla	7	6.4	5 (71.4)	2 (28.6)	
Mandible	9	8.2	4 (44.4)	5 (55.6)	
Buccal	10	9.1	6 (60.0)	4 (40.0)	
Floor of mouth	18	16.4	15 (83.3)	3 (16.7)	
Hard palate	1	0.9	1 (100)	0 (0.0)	
Differentiation					0.03
Well, moderately	98	89.1	66 (67.3)	32 (32.7)	
Poorly	12	10.9	4 (33.3)	8 (66.7)	
Mode of invasion					0.40
1–3	95	86.4	62 (65)	33 (32.7)	
4C, 4D	15	13.6	8 (53.3)	7 (46.7)	
Vascular invasion					0.04
v(−)	101	91.8	62 (65)	33 (34.7)	
v(+)	9	8.2	2 (22.2)	7 (77.8)	
Lymphatic vessel invasion					0.03
ly(−)	92	83.6	63 (68.5)	29 (31.5)	
ly(+)	18	16.4	7 (38.9)	11 (61.1)	
Perineural involvement					0.55
ne(−)	107	97.3	67 (62.6)	40 (37.4)	
ne(+)	3	2.7	3 (38.9)	0 (0.0)	
Recurrence					0.01
(−)	73	66.4	53 (72.6)	20 (27.4)	
(+)	37	33.6	17 (46)	20 (54.1)	

*ECOG PS* Eastern Cooperative Oncology Group performance status, *v* vascular invasion, *ly* lymphatic vessel invasion, *ne* perineural involvement

### Associations between the GalNAc-T3 expression and the clinicopathological variables and pattern of recurrence

In order to identify associations between the GalNAc-T3 expression (weakly vs. strongly positive) and the clinicopathological characteristics of the study cohort, the variables were categorized as shown in Table 3. Consequently, a GalNAc-T3 strongly positive expression was found to be significantly or borderline (in)significantly associated with poor differentiation (*P* = 0.03), the presence of v (*P* = 0.04) and ly (*P* = 0.03) and recurrence (*P* = 0.01) or an older age (*P* = 0.05) in all tumors (Table 3 and Supplementary Fig. 2), respectively. In contrast, there were no significant differences between the patients with weakly and strongly positive GalNAc-T3 ESOSCC expression patterns in terms of gender, ECOG PS, smoking, alcohol use, tumor T stage, location, mode of invasion, and presence of ne (*P* > 0.05) (Table 3). In particular, a strong GalNAc-T3+ expression was evident in v(+) and ly(+) (v; case no. 25, ly; case no. 2 in Supplementary Table 1) representative OSCC components, as shown on EVG and D2-40 staining, respectively (Supplementary Fig. 2).

Additionally, in order to elucidate the correlations between the GalNAc-T3 expression (weakly vs. strongly positive) and the pattern of recurrence in the study cohort, the variables were also categorized as presented in Supplementary Table 2. A strongly positive GalNAc-T3 expression was revealed to be borderline (in)significantly associated with “subsequent regional lymph node metastasis” (*P* = 0.05), whereas there were no significant differences in the patterns of “local recurrence” and “locoregional recurrence” (*P* > 0.05) (Supplementary Table 2).

**Fig. 4:**
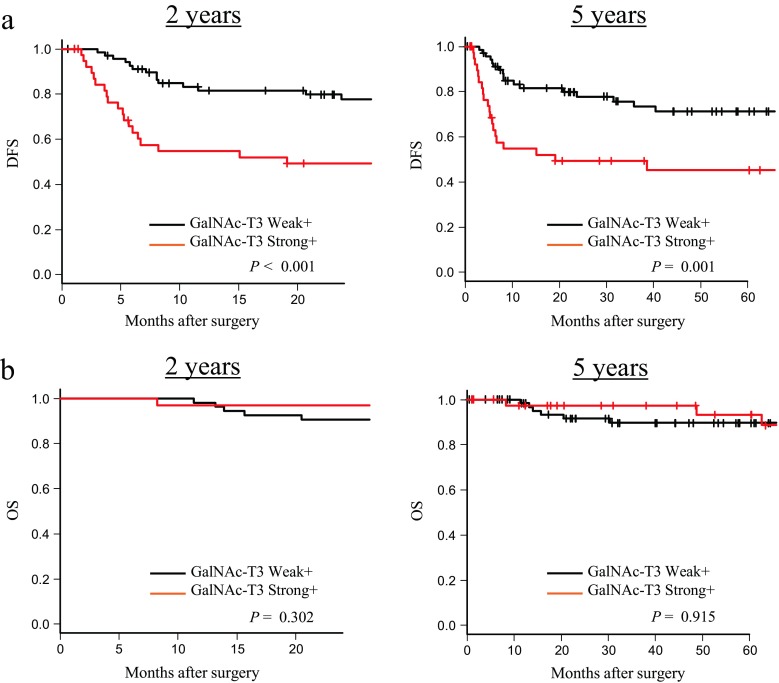
A strong GalNAc-T3-positive expression was associated with a significantly shorter postoperative DFS, but not OS, in the ESOSCC patients, especially within the first 2 years postoperatively. **a** Kaplan–Meier curves for DFS in the patients with ESOSCC within the first 2 (*left*) and 5 (*right*) years after surgery according to the GalNAc-T3 expression. **b** Kaplan–Meier curves for OS in the patients with ESOSCC within the first 2 (*left*) and 5 (*right*) years after surgery according to the GalNAc-T3 expression. *DFS* disease-free survival, *OS* overall survival

According to a Kaplan–Meier analysis, the patients with a strong GalNAc-T3+ expression had a significantly shorter postoperative median DFS (11.8 months) than those with a weak GalNAc-T3+ expression (32.7 months), especially within the first 2 rather than 5 years postoperatively (*P* < 0.001 (2 years) and *P* < 0.01 (5 years), respectively, Fig. 4a). However, there were no significant differences in postoperative OS between the two groups of ESOSCC patients with a weak (median = 62.5 months) and strong (median = 53.7 months) GalNAc-T3+ status (*P* = 0.302 (2 years) and *P* = 0.915 (5 years), Fig. 4b).

### A strong GalNAc-T3+ expression represents a significant independent DFS indicator for ESOSCC by a Cox proportional hazards model

A univariate analysis showed tumor differentiation (poorly), mode of invasion of 4C and 4D, the presence of v and ly, and a strong GalNAc-T3+ expression to be significant predictors of a worse survival (*P* = 0.03, 0.001, 0.01 and <0.001 and <0.001, respectively). Furthermore, a multivariate analysis demonstrated that, after correcting for confounding variables, a strong GalNAc-T3+ expression only remained an independent poor indicator for DFS (*P* = 0.03), whereas a more aggressive mode of invasion and the presence of ly were borderline (*P* = 0.05 and 0.05, respectively) (Table 4).

**Table 4:** Univariate and multivariate analyses of survival (DFS) among the 110 patients with ESOSCC according to the clinicopathological variables and the strong GalNAc-T3 expression

Variables	No. of patients (%)		Univariate			Multivariate		
	Disease-free	Disease	HR	95 % CI	*P* value	HR	95 % CI	*P* value
Age								
≦60	22 (68.8)	10 (31.3)	100					
>60	51 (65.4)	27 (34.6)	1.26	0.61–2.62	0.53			
Sex								
Female	31 (63.3)	18 (36.7)	1.00					
Male	42 (68.9)	19 (31.1)	0.85	0.45–2.30	0.25			
ECOG PS								
0–1	64 (64.0)	36 (36.0)	1.00					
2–4	9 (90.0)	1 (10.0)	0.31	0.04–2.30	0.25			
Smoking								
(−)	34 (66.7)	17 (33.3)	1.00					
(+)	39 (66.1)	20 (33.9)	1.08	0.56–2.06	0.82			
Alcohol								
(−)	22 (68.8)	10 (31.3)	1.00					
(+)	51 (65.4)	27 (34.6)	1.55	0.79–3.00	0.20			
T stage								
T1	42 (71.2)	17 (28.8)	1.00					
T2	31 (60.8)	20 (39.2)	1.53	0.80–2.91	0.20			
Tumor location								
Maxilla, mandible, buccal	23 (65.7)	12 (34.3)	1.00					
Tongue, floor of mouth	50 (66.7)	25 (33.3)	1.20	0.50–2.40	0.82			
Differentiation								
Well, moderately	68 (69.4)	30 (30.6)	1.00			1.00		
Poorly	5 (41.7)	7 (58.3)	2.53	1.11–5.79	0.03	0.69	0.19–2.54	0.58
Mode of invasion								
1, 2, 3	68 (71.6)	27 (28.4)	1.00			1.00		
4C, D	5 (33.3)	10 (66.7)	3.29	1.59–6.82	0.001	3.23	1.02–10.6	0.05
Vascular invasion								
v(−)	70 (69.3)	31 (30.7)	1.00			1.00		
v(+)	3 (33.3)	6 (66.7)	3.54	1.47–8.52	0.01	2.25	0.87–5.78	0.09
Lymphatic vessel invasion								
ly(−)	68 (73.9)	24 (26.1)	1.00			1.00		
ly(+)	5(27.8)	13 (72.2)	3.96	1.98–7.91	<0.001	2.18	1.01–4.71	0.05
Perineural involvement								
ne(−)	71 (66.4)	36 (33.6)	1.00					
ne(+)	2 (66.7)	1 (33.3)	0.87	0.12–6.34	0.89			
GalNAc-T3 expression								
Weak+	53 (75.7)	17 (24.3)	1.00			1.00		
Strong+	20 (50.0)	20 (50.0)	2.81	1.47–5.37	0.002	2.23	1.06–4.80	0.03

*v* vascular invasion, *ly* lymphatic vessel invasion, *ne* perineural involvement

## Discussion

The current study showed for the first time that an immunohistochemically cytoplasmic strong GalNAc-T3-positive expression is a powerful and independent negative indicator of DFS and potential worse outcome in patients with postoperative ESOSCC, especially within the first 2 years after surgery. It is well known that recurrence in ESOSCC patients after curative surgery remains a significant problem and can significantly affect their clinical postoperative course and survival [6]. The biological aggressiveness of ESOSCC is reflected by the capability of carcinoma to recur, even in small OSCC lesions that are considered to have a relatively good prognosis [in

Collectively, our present data are in agreement with the findings of previous studies of several other epithelial cancers [11, 15–24]. For example, kidney epithelial cancer has recently shown a significantly upregulated GalNAc-T3 expression closely associated with more aggressive tumor characteristics and worse prognosis [11]. The present cohort study demonstrated GalNAc-T3 to be a powerful and independent negative indicator of DFS, i.e., a novel marker of recurrence, in patients with postoperative ESOSCC. As to the OS, there were no apparent correlations with a strong GalNAc-T3+ expression of ESOSCC in our study, with the limitations of assessing an unlikely large cohort at a single institution. In fact, all collected patients had surgically resected T1–2N0 ESOSCCs with a more favorable prognosis, showing a 5-year survival rate of 90.9 % compared with that of up to 86 % reported in previous studies [6]. Further follow-up with a larger cohort is therefore needed to confirm the intriguing relationships between a strong GalNAc-T3+ expression and poor outcome in postoperative ESOSCC patients.

Our findings indicate for the first time that a strong GalNAc-T3+ expression in ESOSCC is significantly closely correlated with poor differentiation and the presence of vessel permeation (v(+) and ly(+)), most likely manifesting as significant invasive/aggressive behaviors. These results also accord with those of our recent study of clear cell renal cell carcinoma [11], that the GalNAc-T3+ expression significantly ameliorates adhesive effects together with a significantly low expression of β-catenin, seemingly leading to severe v(+). Moreover, other groups have focused on modulation of the cell adhesion function by GalNAc-T3 overexpression, which is involved in invasion/metastasis of the ovarian adenocarcinoma via abnormal glycosylation of *O*-glycoprotein mucin 1 (MUC1), potentially accompanied by the downregulated expression of E-cadherin and β-catenin [24]. Taken together, we can support our hypotheses that GalNAc-T3 accelerates ESOSCC invasion/metastasis by affecting epithelial–mesenchymal transition (EMT) at least in part, as previously described [11, 21]. Furthermore, our immunohistochemical examination of ESOSCC tissues showed a strong subcellular cytoplasmic staining pattern for GalNAc-T3 especially at the invasive fronts. Whereas, weakly but, on occasion, diversely positive staining was identified in the adjacent non-carcinomatous squamous epithelium (normal to dysplasia). It is conceivable that GalNAc-T3 potentially plays a critical role in acquired OSCC aggressiveness as well as carcinogenesis through aberrant *O*-linked glycosylation. Since GalNAc-Ts reportedly appear in external secretions into body fluids [37, 38], GalNAc-T3 would be a quantitative soluble marker. In this scenario, these findings lead us to speculate that GalNAc-T3 might be not only a predictive factor of occult metastasis or specific tumor marker for OSCC but also an ideal therapeutic target against ESOSCC, together with minimum risk of side effects. Nevertheless, further in-depth in vitro and in vivo analyses, *including real-time quantitative polymerase chain reaction (qRT-PCR)*, are needed to elucidate these issues.

In conclusion, the present cohort study demonstrated for the first time that a strong GalNAc-T3+ expression is an independent, novel, and powerful marker of a worse DFS in ESOSCC patients, especially within the first 2 years, even after curative surgery. Patients with ESOSCC showing strong GalNAc-T3+ expression should be followed up very carefully. Therefore, physicians should assess the value of this critical ESOSCC-specific biomarker, GalNAc-T3, as a useful parameter for clinical management, particularly in the early postoperative phase.

## Electronic supplementary material

Below is the link to the electronic supplementary material.

Supplementary Table 1 (DOC 157 kb)

Supplementary Table 2 (DOC 41 kb)

Supplementary Fig. 1 Immunofluorescent analysis of GalNAc-Ts in HSC-2. Immunofluorescence staining of the HSC-2 cells showed a specific, cytoplasmic perinuclear expression of GalNAc-T3 (*green-stained*), but not in the nuclei (*blue-stained* by Hoechst). In contrast, a much weaker or absent cytoplasmic expression of other GalNAc-Ts (-T1, -T2, -T4 and -T6) was detectable in the same cell line. Representative images are shown (Original magnification: ×400). *Bar* = 20 μm. (PPTX 3,282 kb)

Supplementary Fig. 2 A strong GalNAc-T3+ expression in the ESOSCC cells exhibited a significantly close relationship with a pathological ly(+) and v(+) potential, manifesting as more invasive/aggressive characteristics. Representative pictures for H&E, EVG and the immunohistochemical analyses of GalNAc-T3 and D2-40 in the areas of vascular (v; case no. 25) and lymphatic (ly; case no. 2) invasion among the deeply involved ESOSCC components (Original magnification: ×100; inset: ×400). EVG and D2-40 staining very clearly revealed elastic fibers in the vascular medial wall (v(+)) and lymphatic endothelium (ly(+)). Each inset provides a representative image of ESOSCC cells with a cytoplasmic staining pattern of GalNAc-T3 on high-power view. Bars = 200 μm (×100) and 20 μm (×400). *H & E* hematoxylin and eosin, *EVG* Elastica van Gieson, *v* vascular invasion, *ly* lymphatic vessel invasion (PPTX 5,554 kb)
