# Supplementary material for: Strong expression of polypeptide N-acetylgalactosaminyltransferase 3 independently predicts shortened disease-free survival in patients with early stage oral squamous cell carcinoma
Source: Tumour Biol. 2015 Aug 22;37(1):1357–68. doi: 10.1007/s13277-015-3928-7 (PMC4841842; doi:10.1007/s13277-015-3928-7)
Supplement: Supplementary file 1 — (DOC 157 kb) [file 13277_2015_3928_MOESM1_ESM.doc]

**Supplementary Table 1. Detailed relationship between the GalNAc-T3 expression and each variable.**

**
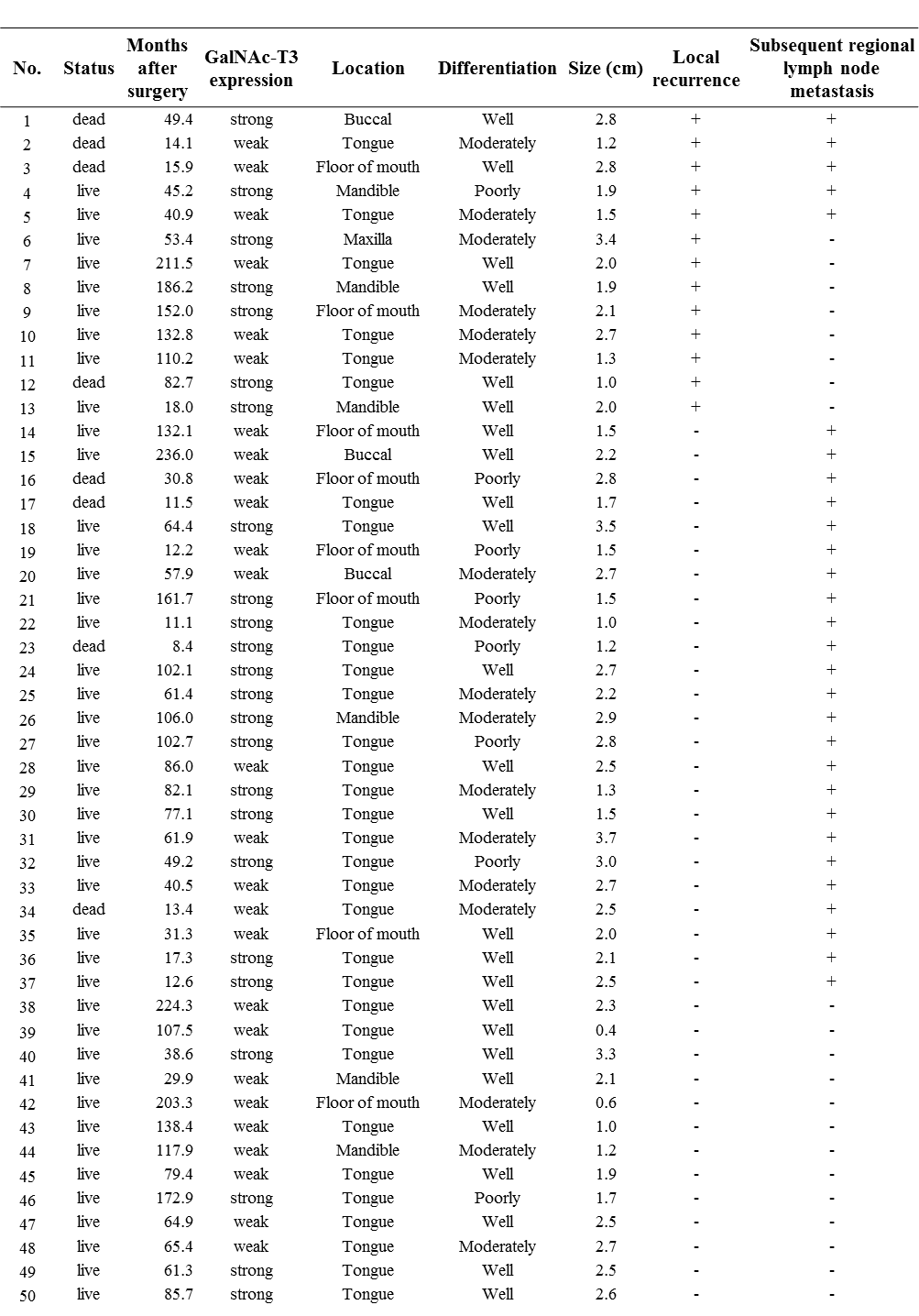
**

**
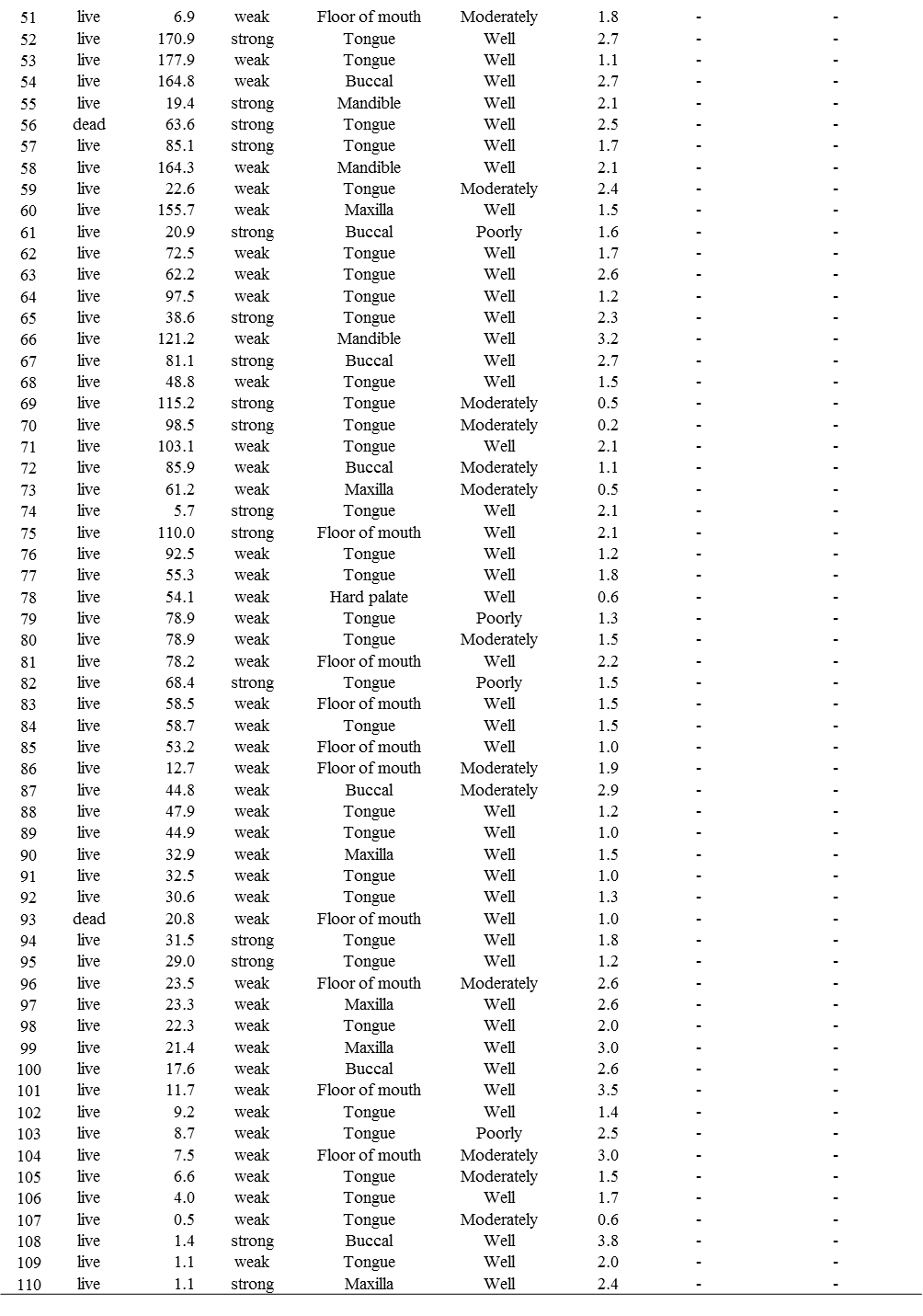
**

v: vascular invasion. ly: lymphatic vessel invasion. ne: perineural involvement.
