# Supplementary material for: Strong expression of polypeptide N-acetylgalactosaminyltransferase 3 independently predicts shortened disease-free survival in patients with early stage oral squamous cell carcinoma
Source: Tumour Biol. 2015 Aug 22;37(1):1357–68. doi: 10.1007/s13277-015-3928-7 (PMC4841842; doi:10.1007/s13277-015-3928-7)

**Supplementary Table 2. Detailed correlations between the GalNAc-T3 expression and the pattern of recurrence.**


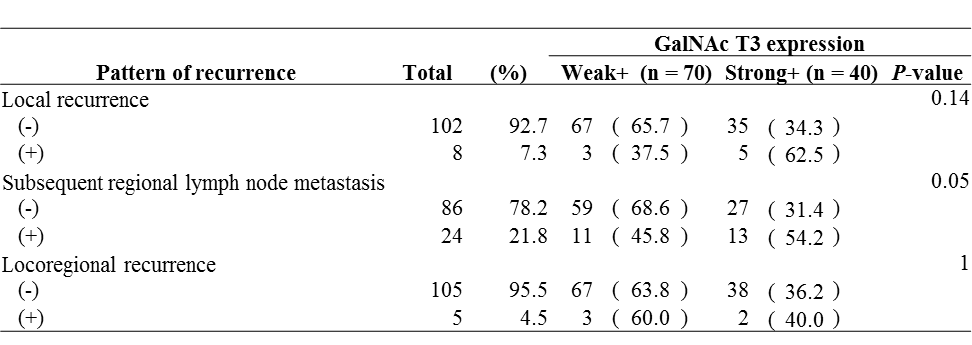

Supplement: Supplementary file 2 — (DOC 41 kb) [file 13277_2015_3928_MOESM2_ESM.doc]
