# Supplementary figures and images for: Strong expression of polypeptide N-acetylgalactosaminyltransferase 3 independently predicts shortened disease-free survival in patients with early stage oral squamous cell carcinoma
Source: Tumour Biol. 2015 Aug 22;37(1):1357–68. doi: 10.1007/s13277-015-3928-7 (PMC4841842; doi:10.1007/s13277-015-3928-7)

## Slide 1
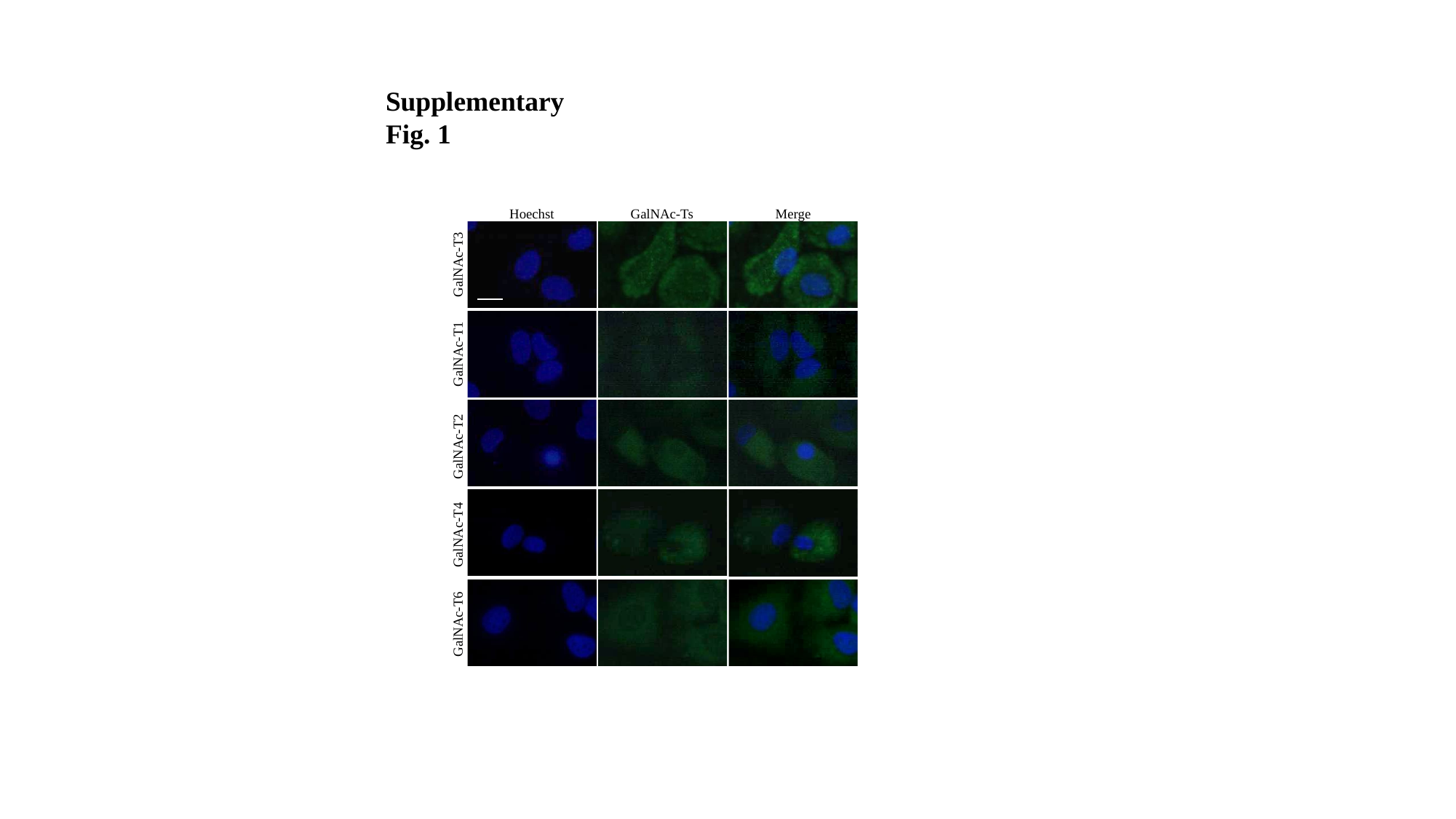

Supplementary
Fig. 1
Hoechst
GalNAc-Ts
Merge
GalNAc-T3
GalNAc-T1
GalNAc-T2
GalNAc-T4
GalNAc-T6

Supplement: Supplementary file 3 — Immunofluorescent analysis of GalNAc-Ts in HSC-2. Immunofluorescence staining of the HSC-2 cells showed a specific, cytoplasmic perinuclear expression of GalNAc-T3 (green-stained), but not in the nuclei (blue-stained by Hoechst). In contrast, a much weaker or absent cytoplasmic expression of other GalNAc-Ts (-T1, -T2, -T4 and -T6) was detectable in the same cell line. Representative images are shown (Original magnification: ×400). Bar = 20 μm. (PPTX 3,282 kb) [file 13277_2015_3928_MOESM3_ESM.pptx]

## Slide 1
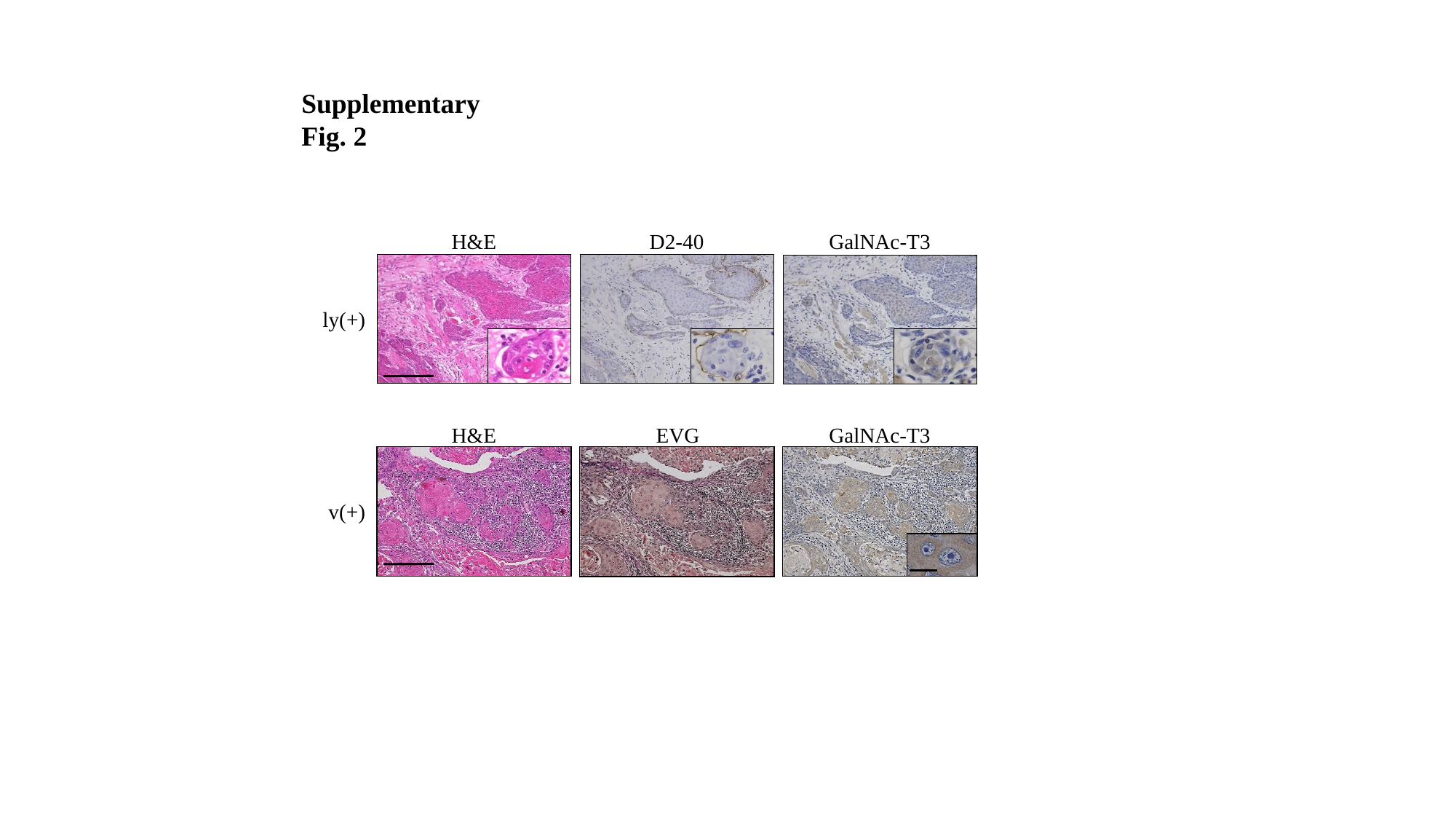

Supplementary
Fig. 2
H&E
D2-40
GalNAc-T3
ly(+)
H&E
EVG
GalNAc-T3
v(+)

Supplement: Supplementary file 4 — A strong GalNAc-T3+ expression in the ESOSCC cells exhibited a significantly close relationship with a pathological ly(+) and v(+) potential, manifesting as more invasive/aggressive characteristics. Representative pictures for H&E, EVG and the immunohistochemical analyses of GalNAc-T3 and D2-40 in the areas of vascular (v; case no. 25) and lymphatic (ly; case no. 2) invasion among the deeply involved ESOSCC components (Original magnification: ×100; inset: ×400). EVG and D2-40 staining very clearly revealed elastic fibers in the vascular medial wall (v(+)) and lymphatic endothelium (ly(+)). Each inset provides a representative image of ESOSCC cells with a cytoplasmic staining pattern of GalNAc-T3 on high-power view. Bars = 200 μm (×100) and 20 μm (×400). H & E hematoxylin and eosin, EVG Elastica van Gieson, v vascular invasion, ly lymphatic vessel invasion (PPTX 5,554 kb) [file 13277_2015_3928_MOESM4_ESM.pptx]
